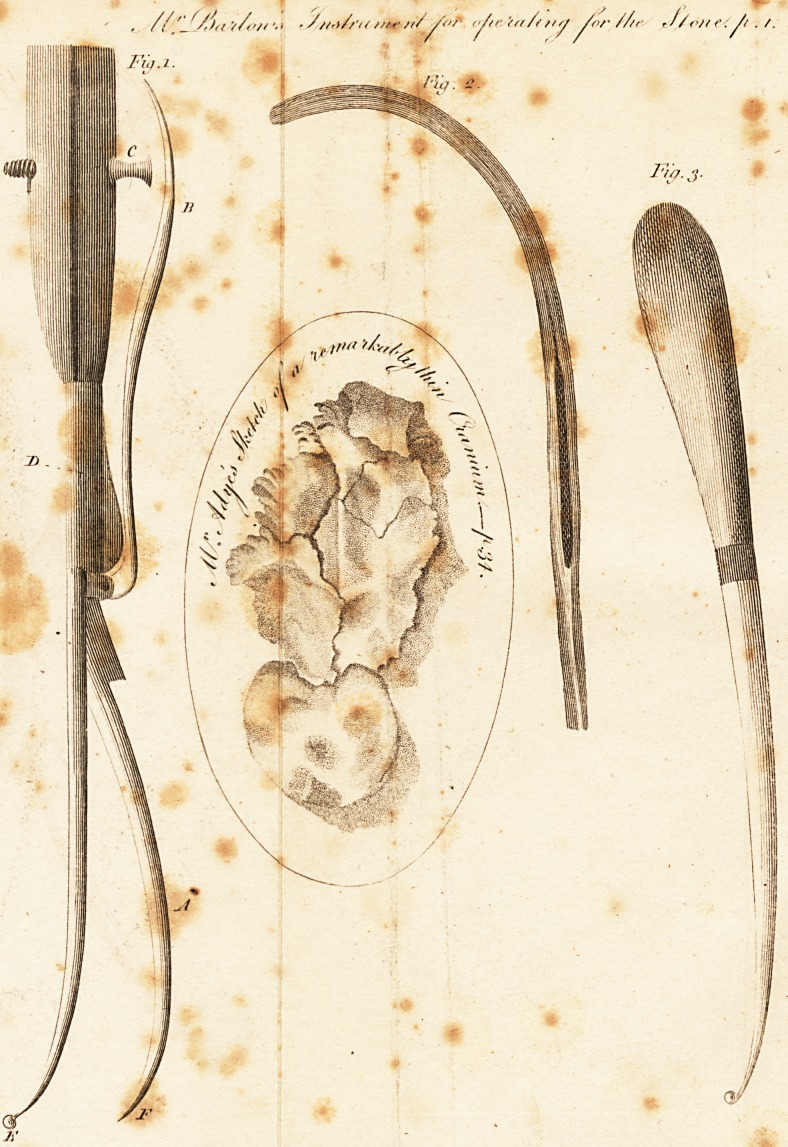# Practical Observations on the Operation for the Stone

**Published:** 1804-01-01

**Authors:** 

**Affiliations:** Blackburn, Lancashire


Medical and Phyfical Journal.

VOL. XI.] JANUARY 1, 1804. [NO. LIX.

Printed for R. PHILLIPS, by W. Thorne, Red Lion Court, Fleet Street, London.

### Practical Observations on the Operation for the Stone. *Communicated by* Mr. Barlow, *of Blackburn, Lancashire.*

[ With Engravings. ]

THE extraction of the stone from the human bladder, by the operation of Lithotomy, has, from the earliest periods of Surgery to the present day, been ranked amongst the most difficult and dangerous operations, and has particularly engaged the attention of many ingenious men; and a multiplicity of instruments have been invented, to facilitate its performance. Annexed to the following remarks, I have sketched an outline of the bistoire caché,* with a beak and staff, with a contracted groove for the beak of the bistoire to slide in. Should this additional invention to this instrument, together with the observations contained in the following pages, meet your approbation, they are at liberty for insertion in your valuable Journal; and if the inexperienced operator be hereby prevented from error, my intention will be fulfilled.

The stone in the urinary bladder is a disease, which neither sex, nor age, are wholly exempt from, nevertheless men are much more liable to this complaint than women, owing to the peculiar conformation of the urinary organs.

Notwithstanding the assiduity bestowed by the Faculty, in endeavouring to find out a specific solvent for the stone, it is truly lamentable to observe, that, hitherto, the most ardent attempts have proved ineffectual. Though stones are more frequently found in the bladder and kidneys than other parts of the system, nevertheless there are numerous instances recorded by writers, of stones being found in almost

* We are indebted for the original contour of the bistoire caché, to Frere Cofme, a French furgeon.

almost every part of the human body.* If a stone be once formed of a magnitude too great to be expelled naturally by the urinary channel, it is highly probable that the art of Medicine can effect no salutary change on the stone, sufficient to destroy its texture completely, though in some particular constitutions, and periods of the complaint, I am disposed to believe, that certain medicines may so far counteract the disposition to the formation of calculi, as to prevent in some degree their further growth for a certain time, and may also produce a change in the bladder, sufficient to appease the consequent irritability excited by the existing stone; it yet remains a task for the chemical philosopher, to point out hereafter a menstruum sufficiently powerful to decompose the urinary calculi in the bladder, without injury to that viscus; and it is to be ardently hoped, that the time is not far distant, when this arcanum will be disclosed. It would be foreign to my present purpose, to enumerate the various medicines, which have at different periods been administered to abate the distressing symptoms of this disease, or otherwise intended to destroy the texture of the calculi in the bladder; it is sufficient in this place to observe, that the hazard attending the exhibition of lithontriptics, if not cautiously administered, may prove deleterious to the constitution of the patient; and when entrusted to the empiric, the most pernicious consequences may be dreaded, insomuch that in some instances the patient's health has suffered irreparable injury, and the advantages otherwise derivable from lithotomy have been thereby precluded.

Though most authors have enumerated various symptoms, as being unequivocal marks of the existence of stone, yet these are not to be considered altogether as distinct and infallible guides; some of the symptoms accompanying

* It is to be observed that quadrupeds, and every human being, are liable to calculous concretions being deposited in different parts of the body, and many instances are recorded by authors, of the existence of stones in the bladder and kidnies of different animals of an enormous magnitude. Morgagni relates feveral examples which occurred to him when employed in dissecting dogs; in the bladder of one of these animals he found a stone which weighed a pound and a half.

In the Memoirs of the Royal Academy of Surgery at Paris, there are many instances related of calculous concretions being found in the matrix, by M. Lewis; and in the Journal des Scavans, is a remarkable cafe of the same kind; and whoever wishes to investigate the subjeet more minutely, may peruse the works of the following authors, Marcellus Donatus, Tolet, Ambrose Parey, Hildanus, Le Dran, Hody, Lieutaud, Bailie's Morbid Anatomy, and Friend's History of Physic.

ing stone in the bladder are very similar to other affections of its appendages; such, for instance, are the diseases of the kidneys and prostate gland; and in some instances the mind of the patient has been strongly impressed with a vague idea of the existence of a stone in the bladder, but on a thorough investigation of the malady, it has sometimes been found to originate from some occult disease affecting the vicinity of the bladder, which on a superficial inquiry very much resembled the symptoms so often concomitant on stone in the bladder.

The difficulty and danger attendant on the operation of lithotomy, has been pointed out by most surgical authors, and in the earliest periods of medicine, of which we have any traces of this malady transmitted to us; we find the operation was principally ordained by the antients to be confined to a select class of men, who made it their peculiar business, and who were termed by the Greeks Lithotomi, and are called by the moderns Lithotomists; this we perceive is sufficiently illustrated in the oath of Hippocrates,* and fully accords with this axiom in Horace:

"———— Quod medicorum est

Promittunt medici, tractant fabrilia fabri."

Where, amongst other ingenious precepts set forth therein by this venerable author, I will relate one to elucidate the candour and purity of mind which this father of medicine possessed. "I swear, says he, by Apollo the physician, by Esculapius, by his daughters Hygeia, Panacea, and by all the gods and goddesses of the Heathen Mythology, that cutting for the stone I will not meddle with, but leave it to the operators in that way." Hence it is highly probable, that the mode of operating for the stone, at this early period, was solely confined to a set of empirics, who, in all likelihood, kept it a secret.

Celsus is the first author who has given any particular account of this operation, from which it has derived the appellation of the Celsian or Guidonian method, or Apparatus Minor, or Cutting upon the Gripe;† and was the

* Though Hippocrates, as also some of the less antient writers, have ranked wounds of the bladder as being inevitably mortal; yet many examples are to be found to the contrary, amongst the works of Tulpius, Vanderviel, Paré, Bauhin, Hildanus, Morand, and others.

† This operation of cutting on the gripe, has since the time of Johannes de Romanis, been called cutting with the lesser apparatus, by way of distinction from his new method of operating, named the greater apparatus, or apparatus magnus, from the greater number of instrunents employed in it.

chief mode of operating promulgated during the space of sixteen centuries. Celsus directs the operator to introduce the fore and middle fingers* of the left hand into the anus of the patient, (if a male) and lay his right hand lightly upon the lower part of the abdomen; by this means the stone is brought to rest upon the neck of the bladder; a lunated incision is then to be made in the skin, near the anus, as far as the neck of the bladder, with the horns pointing a little towards the ischia; then, in that part where the bottom of the wound is straiter, under the skin, a transverse wound must be made, by which the neck of the bladder will be cut, and the urinary passage be something larger than the stone; when the opening is finished, the stone comes into view, which, if small, may be pushed forwards, and taken out with the fingers; but, if large, it must be extracted with the uncus or crotchet.† Various conjectures have however been advanced by authors, respecting the exact method in which Celsus operated; and to settle this point in dispute would be a task neither easy nor useful, more particularly when we consider the imperfect state in which anatomy was understood during the period in which this author lived; and it is probable, that this method, as above described, was not the only one of extracting the stone from the bladder in the days of Celsus, though he is silent on that head.‡ The method of operating described by Celsus, though easily effected upon young and spare subjects, is nevertheless attended with considerable hazard by the transverse incision, for if either the vesiculæ seminalis, or the vasa deferentia, be divided, or their excretory ducts wounded, their functions will be destroyed, and a privation of all future procreation will be incurred; and the same consequences ensue as if castration had been performed; and some one or other of these parts, by the semilunar and transverse incision as above directed by Celsus, must be inevitably wounded in the operation,

* P. Egineta was the first writer who directs the operator to use the forefinger only, and this practice is certainly more eligible, particularly in young subjects.

† It is a little surprising that Celsus should limit the operation to those between the age of nine and fourteen years, as it is probable his mode of operating would be equally eligible during the periods of old age and infancy. Paulus differs in some respects from Celsus on this point, for he allows the operation to be practicable both in the middle and sometimes in an advanced age.

‡ Albucasis, an Arabian physician, has given a pretty full account of the operation of lithotomy, particularly in women.

operation, particularly if the stone be of considerable size; these objections have no doubt caused the Celsian method to be almost universally exploded.

The Great Apparatus, (or *Methodus Mariana)* was invented by Johannes de Romanis, and made public by his scholar Marianus, about the year 1535. In this operation a grooved staff is conducted into the bladder along the urethra, the handle of which is to be turned over the right inguen of the patient, by which the concave part of the staff will appear prominent in the urethra, on the left side of the perineum, in which position it must be secured by an assistant with one hand, while he raises up the scrotum with the other; the operator then makes an incision from the termination of the scrotum, and on the left side of the perineum, to within an inch of the anus; the urethra is next to be divided, from its bulb to the commencent of the prostate gland; and this is usually effected by turning the back of the knife towards the anus, and finishing the incision in the direction towards the scrotum. The urethra being thus laid open, a conductor, or blunt gorget, is to be introduced into the bladder, and the staff withdrawn; the neck of the bladder is then to be dilated sufficiently to allow the extraction of the stone, and various instruments have been invented for this purpose. This operation appears liable to many objections, for neither the prostate gland, nor the neck of the bladder, are divided by the scalpel; and the dilating of these parts sufficiently for the purpose of extracting the stone, must unavoidably produce considerable laceration, and not unfrequently irreparable injury to the patient. The more antient method of cutting upon the gripe might probably be executed with more ease, and greater safety to the patient, than by the great apparatus; provided the operator, after fixing the stone on the left side of the perineum, made the incision in the manner directed in the lateral operation.

### *Of the High Operation, or Apparatus Altus.*

This operation was made known in the year 1561, by Pierre Franco, who performed it with success, on a boy of two years old, at Lausanne, in Switzerland; for on finding the stone too large to be extracted by the operation in the perineum, he had recourse to the high operation, as a method of necessity, rather than choice, and attributes his success more to chance than art, and earnestly dissuades his professional brethren from imitating the practice, and wholly abandons the operation in future himself; since which time no examples are recorded of the high operation, till about the year 1719, when Mr. Douglass and Cheselden adopted this method in England, and afterwards the same mode of operating was practised by Morand, in France.

It is the middle of the anterior part of the bladder, where the incision is to be made in performing this operation; but except this viscus be considerably distended, which is very seldom the case when affected with this malady, there is much danger of wounding the peritoneum; and to avoid this, different methods have been proposed and adopted, with a view of distending the bladder to facilitate the operation; some authors recommend air forced therein by a pair of bellows,* others prefer injections of warm water prior to the operation, and to be retained in the bladder, by means of compression upon the urethra; another method has been adopted of allowing the patient a liberal use of diluent liquors for some time before the operation, and securing the urine in this viscus with a ligature upon the urethra; this last mentioned mode of distending the bladder appears to me the least objectionable, as there is certainly much risque incurred by whatever means that organ is artificially distended, for if the tone of the muscular fibres be destroyed, all hopes from the operation of lithotomy may be abandoned.†

In order however to identify the plenitude of the bladder, in its natural state, the finger of one hand of the surgeon may be introduced into the anus of the patient, and the other hand be laid on the hypogastrium; and by an alternate pressure from these two points, the operator may form a tolerable idea of the capacity of this viscus, and probably enable him to ascertain, in some degree, the size of the stone; some knowledge may also be acquired of the internal capacity of the bladder, by observing the quantity of urine discharged by the patient at different intervals before the operation; and this may in some measure tend to direct the operator of the certitude with respect to the quantity of liquor it is proper to inject or restrain in the bladder, without proving injurious.

When the operation is to be performed, and every thing in

* It is more than probable that Franco did not distend the bladder, either by injection or inflation, upon the child, on whom he cut after the high operation.

† Cheselden relates a fatal instance, where the bladder was burst by injecting too much water into it.

in readiness, an incision is to be made in a line with the linea alba, and a little to one side, beginning about four inches and a half above the ossa pubis, (if an adult) and bringing it down to the junction of the symphisis; the integuments being thus divided, the incision must be carried between the recti and pyramidal muscles, down to the bladder, which viscus is then to be laid open in a direction parallel with the external wound, but not quite to the same extent; the bladder is then to be punctured, and the ligature on the penis at the same time removed, lest the water be extravasated in the cellular texture; the stone is then to be extracted by manual assistance, and the wound of the integuments secured by a few sutures, leaving a small opening at the part next the pubis for the purpose of permitting any discharge which may accompany the progress of the cure.

It is evident that larger stones may be extracted by this operation, and with greater facility to the surgeon than by any other means yet adopted, and various have been the opinions of authors, respecting the advantages and disadvantages attendant on the high operation, when compared with the other methods; nevertheless, when the stone is of a magnitude too great to be extracted by the lateral operation, the high operation undoubtedly presents a resource, confirmed by the authority of authors of the greatest veracity, such for example are Franeus, Winslow, Colot, Middleton, Cheselden, Greenfield, Garengeot, Thornhill, and Heister.*

Morand, in a dissertation on the high operation for the stone, says, "Though I am convinced of the advantages and facility of this operation compared with the method of Marianus, I think it would be imprudent to undertake it upon all without distinction; perhaps it may have been their performing it upon all indifferently, that has a little discredited it in England;" and he further remarks, "That the high operation is the only sure method of extracting large stones and those which are contained in some particular bag† of the bladder, as has been often seen."

To

* This last mentioned author records a case wherein he failed in extracting a fragment of a stone by cutting in the perineum, and was afterwards under the necessity of having recourse to the high operation.

† Though the existence of stones being found encysted, or adherent in the bladder, has been much doubted by some writers, yet many instances of such cases having occurred, are related by the following authors, Schenkius,

 Mercurialis,

To shew further the high estimation with which this operation was at one time held, I will transcribe a passage from Douglass, in the Appendix to his Translation of Morand's Dissertation on the Stone. "From all which I think I may justly infer, that we ought to make the high operation in all cases from five to fifty years of age, when the patient is otherwise in a good state of health, because experience, the best of all arguments, shews that such patients stand a better chance of living after this than any other method; and above all, have no ground to fear being plagued during life with a fistula, incontinency of urine, or of being made impotent by it; one or all of which often succeeds the other operations."

It appears evident from these testimonies, and the history of some remarkable cases, that the high operation would be more safe and eligible, under some particular circumstances, than the lateral method, and particularly where there is ground to believe the stone to be either very large, or a number of stones cohering to each other, or where the bladder is divided into two or more bags or cavities; such cases, for example, are related by different authors. Hildanus mentions the case of a young man of twenty years of age, from whose bladder he extracted a stone which weighed 22 ounces, and had the figure of a cupping glass; the patient however died in the hands of the operator. See Cent. iv. Obs. 5; also Cent. iv. Obs. 50.

It is asserted that Collot Germain, an eminent French surgeon, performed the operation of lithotomy with success upon a criminal in the reign of Louis XI. King of France, and though historians are divided in opinion with respect to his mode of operating, yet there is some reason to suppose he adopted, in this instance, the high operation, because, among other circumstances, he mentions the reduction of the intestines, and the suture of the abdomen. If this was the case, it is probable that he was the first Lithotomist who attempted the section above the pubis; and may we not from hence, with more propriety, date the epocha of the high operation to this incident of the criminal,

---

Mercurialis, Fernelius, Bauhin, Barbette, Holerius, Heister, Sharp, and Dr. Preston, in the Philosophical Transactions, and also the Memoirs of the Royal Academy of Surgery at Paris. M. Desault in the Parisian Chirurgical Journal, has given a plate representing an instrument he has invented, called a Kystitome, which appears well calculated for the purpose of facilitating the extraction of encysted stones; and I am informed it has been used in this country with success.

minal, as recorded by Collot, than to that related by Franco on the child at Lausanne in Switzerland; for Collot lived near a century antecedent to that of Franco, who has hitherto been universally applauded as the promulgator of the Hypogastric section.

Louis the Eleventh reigned from the year 1461 to 1483, and Collot then being one of the first Lithotomists in that kingdom during the reign of this prince, his superior skill in operating is said to have descended to his posterity; and Philip Collot, a descendant of the same family, made some valuable improvements in the apparatus and method of operating.

### *Of the Lateral Operation.*

The epocha in which the lateral operation was first promulgated was in the year 1697, by an ecclesiastic, whose name was Frere Jaques; he arrived, says Dionis, at Paris, as a sort of monk, in the habit of a rocolet, with this difference only, that he wore shoes, and instead of a cowl had a hat. He assumed the name of Brother James, and appeared plain and ingenuous; his diet was very sober, he lived on pottage and bread only; he had no money, and never asked any more than a few sols to pay for the setting of his instruments and mending his shoes. He formed to himself a religion according to his own fancy, backed with vows, the liberty of dispensing with which he left to his ordinary at pleasure. On coming to Paris, he produced a number of certificates as a testimony of his dexterity in operating in several provinces of France, which being presented to the surgeons of l'Hotel Dieu and de la Charité, his offers were rejected, as not being customary to expose hospital patients to the risk of experiments; nevertheless, a dead body was procured, into whose bladder a stone was conveyed, and which Frere Jaques extracted in the presence of many surgeons; but his method not being approved of, and Jaques finding himself coldly received by the physicians, resolved to leave Paris and retire to Fontainbleau, where he met with encouragement; for being allowed to operate on a boy from Versailles, and the operation succeeding, he thereby gained much applause; it is however extraordinary he used no preparatory regimen, or medicines, to his patients, nor troubled himself after the operation with any application to the wound, except a little oil and wine, on which he relied for the cure. It is said he was so intrepid in his operation, and negligent with regard to his patients, that when he was entreated by them for his more more particular attention, he would answer, it is sufficient that I have extracted the stone, God himself will cure the wound. The reputation of Frere Jaques spread rapidly, and he afterwards returned to Paris, where he continued to practice gratuitously, and with tolerable success, for some time; but the excruciating tortures which the Marshal de Lorye suffered from his rash treatment, gave cause for the chief surgeons to investigate more particularly into his mode of operating; and this enquiry turning out unfavourably for Jaques, the consequence was that his reputation as a lithotomist was lost, and he became afterwards branded with ignominy, insomuch that even his partisans considered him a rash and ignorant operator; he therefore quitted France, and travelled through Germany in the capacity of a lithotomist. But Mr. Sharp appears to have a more favourable opinion of his mode of operating than most authors, for in his Treatise on the Operations of Surgery, he says, Jaques knew more of lithotomy than is generally imagined; for he saw when in France, a Treatise on this operation, published by Jaques, in the year 1702, wherein his method of operating is described as not being essentially different from the lateral operation as practised in his time; and as a further proof of the success he acquired, there is a certificate annexed to the publication, where it is asserted he had cut thirty-eight patients successively at Versailles, without losing one, and had so much improved his apparatus as to make use of a grooved staff.

The lateral operation, as practised by Frere Jaques, being so generally known, it is unnecessary in this place to enter into a detailed account of his invention; it will be sufficient to give a brief narration of the lateral operation as now practised by most lithotomists. It may not, however, be improper first to recite the boundaries, and parts connected with the operation, as guides to facilitate its performance with greater safety to the patient; these are the symphysis pubis, raphe, tuberosity of the left ischium, anus, and the convex projection of the staff in the perineum. The parts to be avoided in the operation are, the bulb of the urethra, the pudical artery, rectum, and vesicula seminalis. When the patient is properly secured, and every thing in readiness for the operation, the surgeon should introduce the staff and distinctly feel the stone, after which the handle of the instrument must be made to pass over the right groin of the patient, and be secured in the situation by an assistant, in such a manner that the concave part may be evidently felt on the left side of the perineum; perineum; then the surgeon, with the utmost coolness and undaunted firmness, must begin the incision about an inch below the termination of the scrotum, a little under the arch of the ossa pubis, and rather on the left side of the rapha, cutting through the skin and cellular substance, in an oblique direction, for the space of four inches, (if an adult) along the perineum, and in an equal line betwixt the tuberosity of the left ischium and anus, and terminating about an inch below the approximation of these two points; the skin and cellular membrane being divided, the muscles are then to be intersected in a line with the staff, and external incision; the groove of the staff should then be found, by pressing the point of the fore finger of the left hand against the sulcus, as a guide for the point of the knife; the membranous part of the urethra is then to be divided, and this may be effected with greater safety by turning the back of the knife downwards; and the index of the operator's left hand being made to press down the rectum, whilst the cutting edge of the knife divides the membranous part of the urethra upwards, along the sulcus of the staff, the whole length from near the commencement of the prostate gland to the bulb of the urethra; the back of the gorget is then to be conducted into the groove of the staff, about an inch from the prostate gland; the operator then takes hold of the handle of the staff with his left hand, which he elevates to nearly a right angle with the body of the patient; he then raises himself from his seat, and, at the same time, with the staff in the left hand, and the gorget in the right, with its beak in the sulcus, he makes a uniform pressure with one hand against the other, and gently pushes the gorget along the groove, at the same time depressing the handle, till it has evidently passed into the bladder; the staff is then to be gently withdrawn in a direction, with the concave part towards the pubis of the patient; the fore finger of the operator's right hand is then to be passed into the bladder, upon the gorget, and if the stone can be thereby detected, the gorget may then be withdrawn, and the middle finger of the same hand be introduced in its place; the stone may generally be extracted by the combined efforts of the two fingers thus acting as forceps; if this however cannot be accomplished, the forceps must then be cautiously introduced along the fingers into the bladder, which are then to be withdrawn, and the stone searched for with the forceps, and extracted according to the precepts so minutely described by authors.

It is to be observed, that in emaciated subjects, and where the operator is perfectly acquainted with the anatomy of the parts connected in lithotomy, I am persuaded the division of the prostate gland, and the necessary portion of the neck of the bladder, may be much more easily and safely executed, with either the common scalpel, or a beak pointed bistory, than the gorget; and particularly in cases where the stone is fixed at the neck of the bladder, and the natural entrance into that viscus is so much blocked up, that the complete introduction of the staff is impracticable. Thus circumstanced, the operation may be best effected by the surgeon passing the staff to the place of obstruction, and then making an incision with the scalpel into the urethra on its point, and after withdrawing the staff, the beak pointed bistory (as represented in the annexed plate) is to be gently passed along the urethra, by the side of the stone, into the bladder, and the division of the prostate gland and neck of the bladder be made by withdrawing the instrument, and executing the division of the parts in a lateral direction, as hereafter specified in the use of the improved bistoire caché. For example, the cases requiring this mode of treatment may be found in the Medical Facts and Observations, vol. 8; Medical Records and Researches, London Memoirs, vol. 5, Ingram's Observations in Surgery, Saviard's Surgical Observations, and the Edinburgh Essays and Observations, vol. 3.

I have thus given a brief history of the various methods of extracting the stone in men, which has at different periods of time been sanctioned by cotemporary lithotomists, and it is hoped the chirurgical reader will not accuse me of palpable omission, in not having entered more fully * into the collateral incidental circumstances sometimes attendant on lithotomy; for, though conciseness has been my aim, yet I hope this general outline will be sufficiently illustrative of the subject, as to convey an adequate idea of the utility consequent on the following remarks; I nevertheless flatter myself, that the additional improvement which

* It is my intention, should these pages meet the approbation of the public, on some future occasion, to prosecute the subject still further, by drawing a few practical inferences of the comparative advantages, and disadvantages, attendant on each mode of operating; together with the regimen necessary to be observed; and also observations on the formation and decomposition of calculi in the bladder, with remarks on the operations of Nephrotomy, &c.

which I have made to the bistoire caché, as represented in the third volume of the Medical and Physical Journal, will be sanctioned by most lithotomists; and though I have operated oftener than once successfully with the instrument above alluded to, yet its defects have to me appeared distinctly palpable. The diversity of alteration which the gorget has undergone, with a view to surmount certain difficulties attending its introduction into the bladder, are a sufficient apology for practical novelty; yet it is a matter of doubt, whether the advantages from such a multiplicity of interchangeable discoveries, which have succeeded each other in the apparatus belonging to this operation, have been altogether realized in the same ratio with their numbers; it is however at all times to be wished, that in proportion as the art of surgery improves, the number of instruments will be proportionately retrenched; nevertheless, I have to crave the same indulgence in this respect, in common with others of the profession, who have attempted to add something to the stock of surgical improvement; and if I have failed herein, let it be remembered that I have endeavoured to be useful without prolixity; and perhaps I may at least be entitled to some portion of gratitude from the reader, in having condensed into a narrow compass a number of facts and opinions relative to Lithotomy, which are dispersed amongst the works of a multiplicity of surgical authors, and which I have reason to believe are not always easy of accession to the generality of readers, without considerable expence, and ardent application. The work of the gorget is to divide the prostate gland, and a portion of the neck of the bladder; but if the practical surgeon attentively and impartially views the mechanism of this instrument, and the parts to be divided, and avoided, in the operation, he will not hesitate to determine that its construction is not well adapted to execute the purpose for which it was invented; and it is doubtful whether ever it can be used, even in its most improved state, with perfect safety, unless the bladder be in a state of distension; which is seldom the case when indicative of lithotomy. In operating for the stone in the common way, there is evident danger of the beak of the gorget slipping out of the groove of the staff; and if pushed to the hilt with the customary force, and to the same distance in every individual case, whether the bladder be collapsed or distended, it is evident, that by this rash mechanical impulse, the point of the gorget may pass through through the back part of the bladder, and puncture the rectum; this dangerous circumstance has been known to happen under the guidance of the most celebrated lithotomists; nor can the gorget make so clear a cut as is to be wished; for, as the parts to be divided are movable by pressure, and apt to recede before the point of any instrument so constructed, it is manifestly evident that a compleat section will be thereby difficult to make. The danger which the gorget incurs to the patient, by being pushed precipitately along, and the liability of its either slipping out of the groove of the staff, and penetrating the intestines, can scarcely be pointed out in terms sufficiently cogent to prevent impendent mischief; but the bistoire caché, with the additional beak, is totally exempt from such accidents, and it is not improbable that most of those patients who have died of this operation, when free from any other disease, have suffered from this accident. Vide Benj. Bell's Surgery.

The bistoire caché in common use, I must acknowledge, is considerably less liable to this accident than the gorget, but is in other respects subject to do mischief, owing to the liability of the point of the instrument sliding out of the groove of the staff; and if much force be used (though not absolutely necessary with this instrument) at this period of the operation, and this event take place, the point of the bistoire may do irreparable injury, by being pushed into the rectum, either before, or after having passed into the bladder. On deliberate reflection, I am firmly convinced that these difficulties and dangers attendant on either of these instruments will be obviated by the use of the improved bistoire caché as before mentioned, together with a grooved staff, as represented in the same annexed plate. Their use are as follows: After the operator has introduced the staff with the contracted groove into the bladder, and so far proceeded in the operation as to have completely divided the membranous part of the urethra, he then, with the beak of the bistoire when shut, conducts it into the sulcus of the staff, at the part where the contraction commences, and then gently and without force pushes it along the groove into the bladder; this step of the operation will be generally identified, either by a flux of urine, or the detection of the stone by the point of the instrument; the staff is then to be withdrawn, and the operator may, by moving the bistoire, ascertain the capacity of the bladder, and probably form some idea of the magnitude, situation situation, and number* of stones contained in that viscus; after which the instrument is to be opened to the extent fixed by the screw at the handle, before its introduction, and the cutting edge is then to be turned obliquely outwards and downwards, that the prostate gland may be divided nearly on the left side, and rather in this direction than horizontally lateral; the surgeon then withdraws the instrument in a straight line towards himself, and with a degree of celerity sufficient to execute the division of the prostate gland, and a part of the neck of the bladder, proportionate to the size of the patient, and sufficient to allow a free exit of the stone, and in a manner most likely to promote a speedy union of the parts; the operator should on withdrawing the bistoire, keep in mind the anatomy of the parts which are to be divided, lest by inattention, the erector penis, and probably a branch of the hypogastric artery, named pudica externa, be divided, and the functions of the parts be thereby destroyed; and should the instrument be placed with the cutting edge too much inclining towards the anus, and be withdrawn in that direction, there will be danger of wounding the vesicula seminales, and intestine. By an attention to these directions, the surgeon will be enabled to perform this important part of the operation with greater ease to himself and safety to the patient, than by the use of the gorget.

Many important advantages I am confident will be attained both to the surgeon and patient, by this mode of operating; and I trust it will appear to the ingenious lithotomist, that this instrument, when used with the contracted grooved staff, claims an established preference over any other invention yet made public. There is in the Medical and Physical Journal, a description of an instrument with a plate annexed thereto, intended to supersede the use of the gorget, and invented by Mr. Watt of Paisley, which appears to possess some advantages over this instrument; it

---

* Various instances are recorded by authors, respecting the number of stones occasionally found in the bladder. I had lately a case under my care of a man, near eighty years of age, out of whose urethra I extracted more than twenty stones, some of which were very large; after death, leave was obtained to inspect the bladder, and out of this viscus nineteen stones were extracted, three of the largest were each of the size of a pullet's egg; the bladder was very much diseased, and in some parts was three-fourths of an inch thick; these stones had a very irregular surface, though there was much uniformity in their shape; they were all of a light and chalky nature.—— Hildanus relates the history of a young man who emitted 300 stones by the urethra. Centur. 1, Obs. 69.

it has, however, this imperfection, that by being made nearly straight, its introduction into the bladder along the sulcus of the common staff, will incur some embarrasment to the operator and danger to the patient. Another material objection to this instrument is, that if the prop or cutting part be raised out of the groove, it forms an acute angle, consequently will be liable to cut a greater portion of the neck of the bladder, than is absolutely necessary, but in other respects is not very dissimilar to the one I have been describing.

The apparatus invented and represented by Sir James Earle, in an appendix to his treatise on Lithotomy, has the merit of ingenuity, and may be a guide to the young operator, in determining the line proper for making the external incision, and the division of the urethra, but is not intended altogether to supersede the use of the gorget, therefore it does not become manifestly necessary in this place to elucidate its use; it may be proper, however, to observe, that in the application of this instrument there will be some difficulty in adjusting the parts of the two staffs exactly together, owing to the tendency which the short staff may occasion by a small deviation at the hinge.

Occidit qui non servat.

### *Of the Extraction of the Stone from Women.*

It has been before observed, that the female sex are much less liable to the existence of stone in the bladder than males, chiefly because the neck of that organ in females is not surrounded with the prostate gland as in men, and the cavity of the urethra being much shorter, less curved, and more easy of distention, renders the spontaneous evacuation of calculi through the canal of the urethra a more common occurrence, consequently the operation of lithotomy becomes seldomer necessary in the female than the male; and if we peruse the works of some authors who have treated on the subject, and credit their accounts of the enormous size of stones naturally discharged or extracted from the urethra, we shall be almost led to believe that the operation of lithotomy will very rarely, if ever, become necessary in females. Such, for example, is the case related by Borelli, (Cent. 2, Obs. 22) of a stone coming spontaneously from a female the size of a goose's egg. Kirkringius and Bartholin, each describes one as large as a hen's egg. And for more instances of a similar nature, the reader may consult the works of Celsus, De

Graaf,

Graaf, D. Hieronymus, Tulpius, Middleton, Colet, Morand, and the Philosophical Transactions.

The earthy particles of the urine have the attractive quality of generating calculi, and adhering to any foreign indissoluble substance in the bladder; which, if not timely expelled, may afford a basis for the formation of a stone. Numerous instances are recorded by authors of needles, bodkins, and other extraneous substances, having been thrust up the urethra into the bladder of females, and become a nucleus for a stone. Morgagni relates several cases illustrative of this fact; and one of a man, in whose bladder, on dissection, was found a calculus of a considerable size, and a needle buried in the centre. A similar instance is mentioned in the Edinburgh Medical Essays and Observations, vol. iv. See also Cheselden, Parey, Tolet, and Dionis. Mr. Proby, in the Phil. Trans. No. 260, page 455, relates a case of a bodkin being successfully extracted out of a woman's bladder by the high operation. See also Medical and Physical Journal, vol. 9. Dr. James, in his Medical Dictionary, relates an instance of a gentlewoman being in labour, and attended by a man-midwife. "When I was called in (says he) he told me the stone was so unluckily situated, that it was impossible to deliver her, every pain bringing the stone into the passage; and was of opinion that the stone should be taken away immediately, before she could be delivered. This however I opposed, and the patient was delivered by proper management. Upon searching her about two months afterwards, I found the stone situated partly in the urethra; so that by drawing the edges of the urinary passages aside, I could see it. Upon this she consented to have the stone taken away, and a day was appointed for the operation; but the morning it was to be performed, her courage failed, and she determined not to undergo it. About six weeks after, I was called again, and found she had parted with the stone spontaneously, without much pain; and afterwards she was for some months troubled with an incontinency of urine, which gradually went off, and she recovered perfectly."

Morand relates the case of a girl, eighteen years old, who by the efforts of nature alone, voided on the 29th of October, 1724, a stone from the bladder, weighing four ounces. Notwithstanding these natural advantages which females inherit, we must acknowledge that they are not absolutely exempt from the necessity of the operation of lithotomy; a case sufficiently shewing that manual assistance is sometimes required, is related in the Philosophical Transactions,

Transactsons, No. 209, p. 103, by Mr. Basil Wood; the length of the stone was 3¾ inches; in breadth where largest, is very near 3¼ inches; its thickness 1⅞; and its weight nine ounces and a half averdupois. More examples of this kind might be adduced from good authority, where the stone has proved too voluminous to traverse the urethra, or even by mechanically dilating* the channel by means of the introduction of some foreign substance, or by the more dangerous method with the instrument called Dilatator or Speculum Vesicæ. There is an interesting case recorded by Mr. Bromfield, which terminated successfully, where the ingenious author introduced the closed extremity of the appendicula intestini ceci of a small animal into the bladder of a girl. Into the open end of the appendicula warm water was injected by means of a syringe, and secured externally by a ligature; and by repeatedly twisting the end which was left out, the cervix vesicæ dilated sufficiently to allow the exit of the stone. The different methods adopted by authors of dilating the urethra, may no doubt be practised with safety to a certain extent,† where the circumference of the existing stone, and the canal of the urethra, bear no great disproportion to each other; but on the other hand, where the size of the stone is supposed to present great disparity of proportion to the natural diameter of the urethra; then, to prevent laceration, the ordinary method of dilatation should be abandoned, and recourse had to either the division of the urethra, as hereafter directed, or the high operation, as Falconet, Heister, Douglass, and Morand‡ recommend.

Some authors have, under these circumstances, adopted the

* Prosper Alpinus, in his Medicina Egypt. page 104, mentions a practice adopted by the Egyptians of inflating the urethra to effect the exit of the stone; but I find no successful examples of this method exercised amongst the Europeans.

† This circumstance is sufficiently proved by the testimony of many authors, amongst whom are Greenfield, Tolet, and Hildanus. We have also a remarkable instance of this kind related in the Miscell. Nat. Curios. Obs. Decad. 2, and 10, of a woman being freed from a stone by the dilatation of the urethra, which weighed five ounces and a half. Douglass recommends the extraction of small stones by dilating the urethra with tents of prepared sponge, or gentian root; but, on the other hand, if the stone be large, he proposes the high operation, and distending the bladder with warm water prior thereto.

‡ This last mentioned author says, "As for the woman, I believe, that if the stone be small, the common method is preferable to the high operation; but, if large, the apparatus altus is better than the ordinary method, because of the incontinency of urine, which happens through the laceration, and extravagant dilatation of the sphincter caused by the passage of a large stone."

the method of making an opening directly into the bladder from the vagina, by which rout the forceps are conducted into that viscus, and the stone by that means extracted from the bladder. Gooch relates three successful cases of this mode of extracting the stone; nevertheless, by cutting through the vagina into the bladder, parts become unavoidably injured, which will in all probability either leave a fistulous opening, or produce a cicatrix, in the vagina; and should the patient afterwards become pregnant, it is probable that during the stage of labour, this disagreeable circumstance would either incur a laceration of the parts, or at least prove a material obstacle to delivery; hence it appears that this mode of operating is on this account objectionable, and not likely to become generally practised.

In those unfortunate cases where the stone can be identified, before the operation too far* exceed the ordinary size, it is certainly better to have recourse to the high operation, than hazard the life of the patient by breaking† the stone with the forceps in the bladder, or otherwise being compelled to the dreadful alternative of the compound operation; and this precept will perhaps be thought more applicable to men than women.

The usual method of extracting the stone from women is comparatively easy when contrasted with the operation in men; it consists in passing through the urethra into the bladder a grooved director, or staff, nearly straight, which the operator is to hold firm with his left hand, while with the right he conveys the beak of the cutting director into the sulcus of the staff, and gently passes it along the groove till it has entered the cavity of the bladder; the stone is then to be extracted, either by the fingers of the

 surgeon,

---

* Professor Borichius, and the celebrated Archiater, died in the operation, owing to the stone being so large that it could not be extracted at the perineum. There is a stone reserved in the Charity Hospital at Paris, which weighs 51 ounces; the man from whom it was extracted died during the operation.

† The first accounts we have on record, of breaking or splitting the stone in the bladder, is mentioned by Celsus, the author of which contrivance was named Ammonus, who upon this account, obtained the appellation of Lithotomist, or the Stone-cutter.

We are indebted to Andreas Cruce for the original invention of a pair of forceps, for the purpofe of breaking stones in the bladder; this instrument, we are told, failed of effecting this purpose in the deplorable case of the celebrated Borichius above alluded to.

surgeon, or the forceps, as is practised in the operation in men.

From the shortness of the female urethra, this operation is more safe and easy to execute than in the other sex, yet it will evidently appear much simplified by the use of the bistoire caché, as before recommended in the operation on men. The mode of using the instrument is as follows: The bistoire is to be introduced shut along the urethra into the bladder; and the surgeon having found the stone with the point of the instrument, he then presses down the handle upon the head of the screw, which elevates the cutting edge; and turning the blade towards the left side, and rather obliquely outward, he withdraws the bistoire in a straight direction, and completes the division of the parts in a similar manner as directed in the operations on men. The use of the bistoire caché is so manifestly simple, and possesses those advantages when used upon women, that it will altogether supersede the use of the sound, grooved staff, and gorget, and may be made of any size proportioned to the age of the patient; and it may be also necessary further to mention, that when used on females it may be made either with or without a beak.

*Nov.* 7, 1803.

---

### REFFRENCES TO THE PLATES.

Fig. 1.

A. The bistoire cache, its length, including the blade and handle, ten inches

B. The lever which is connected with the cutting blade, and by raising or depressing the screw C. the blade is made to open or shut, and may by pressure be elevated from the sheath to a distance suitable to the age of the patient.

C. The screw, which may be raised, or depressed, at pleasure; and the crown is the prop which the lever B. is to rest upon when the blade is elevated.

D. A spring which raises the lever B. upwards, and serves to shut the instrument.

E. The beak or knob which is intended to fit the contracted grooved staff, Fig. 2, and the neck of the beak is to be made rather flat, to give stability thereto, and yet so small that it will pass freely along the contracted part, without letting the beak slip out till it arrives at the open point of the staff.

The blade, the point of which, for the space of three lines, is to be left obtuse, to prevent injuring the coats of the bladder.

Fig.

Fig. 2.

A grooved staff left open at the convex part, for the purpose of receiving the beak of the bistoire E. Fig. 1. and also an opening at the point, for the space of half an inch, for the exit of the beak, after having passed the narrowed part of the staff as far as the opening at the point.

Fig. 3.

Represents a bistory, its whole length seven inches, with a beak, or knob, and a small neck, so adapted as to slide freely, either in the groove of the staff, Fig. 2. or the common staff. This instrument appears well calculated to supercede the use of the gorget, when used on women, and may answer other purposes in various surgical cases.

## Figures and Tables

**Fig.1. Fig. 2. Fig. 3. f1:**